# Frequency of Neurological Disorders in Bullous Pemphigoid Patients: A Cross-Sectional Study

**DOI:** 10.1155/2017/6053267

**Published:** 2017-05-24

**Authors:** Sheida Khosravani, Farhad Handjani, Reyhaneh Alimohammadi, Nasrin Saki

**Affiliations:** ^1^Student Research Committee, Shiraz University of Medical Sciences, Shiraz, Iran; ^2^Molecular Dermatology Research Center, Shiraz University of Medical Sciences, Shiraz, Iran; ^3^Dermatology Department, Shiraz University of Medical Sciences, Shiraz, Iran; ^4^Department of Comprehensive Dentistry, University of Texas Health Science Center at San Antonio, San Antonio, TX, USA

## Abstract

**Background:**

Bullous pemphigoid (BP) is an autoimmune subepidermal blistering skin disorder which occurs mostly in the elderly. Several studies have reported an association between BP and neurological disorders (ND).

**Objective:**

The purpose of this study was to evaluate the association between BP and neurological disorders in Iranian patients.

**Methods:**

In this cross-sectional study, 87 patients with BP were enrolled. They were compared to 184 controls. Statistical analysis was done by SPSS statistical software version 19.

**Results:**

Out of 87 patients with BP, 17 (19.5%) had at least one neurological disease. Cerebrovascular accident (CVA) was the most common neurological disease that was seen in 7 patients (8.0%) in the case group and 4 (2.1%) in the control group. The incidence of CVA was significantly different between BP patients and the control group (*P* = 0.022). Dementia was observed in 6 patients in the case group (16.8%) and 2 (1.0%) in the control group. The incidence of dementia was significantly different between BP patients and the control group (*P* = 0.008). In this study, the incidences of Parkinson's disease (*P* = 0.830), epilepsy (*P* = 0.067), and multiple sclerosis (*P* = 0.326) were not statistically significant between the two groups.

**Conclusion:**

The incidence of CVA and dementia in patients with BP compared to the control group was significantly higher.

## 1. Introduction

Bullous pemphigoid (BP) is an autoimmune subepidermal blistering skin disorder which occurs mostly in the elderly. It is associated with circulating autoantibodies against hemidesmosomal proteins BP180 (BPAG2) and BP230 (BPAG1) in the dermoepidermal junction [1]. Clinical features of this disease include subepidermal blisters on urticarial plaques, erythematous, or noninflamed skin. It occurs mostly on the flexural aspects of the limbs and on the trunk [2]. BPAG2 is a transmembrane protein that has a long extracellular domain and is associated with synapse stabilization in the central nervous system. BPAG1 is an intracellular protein which belongs to the plakin family. The role of BPAG1 is to connect the intermediate filament, microtubule, and microfilament cytoskeletal networks with each other and to cell membrane sites. They are also known as scaffolds for signaling proteins that modulate cytoskeletal dynamics. Different isoforms of BPAG1 have been found: BPAGI-e in the skin, BPAG1-a in the nervous system, and BPAG1-b in the striated muscle [3, 4].

The incidence of BP has been estimated to be 4.5 to 14 new cases per million per year [5]. The prevalence is shown to be increasing in some studies [6, 7]. Although the disease is much more common in developed countries, its prevalence is on the rise in Iran [8]. Moreover, the mean age of patients with BP in Iran is lower than in European countries [9].

In recent years, the association of BP and neurological diseases including dementia, stroke, Parkinson's disease, epilepsy, and multiple sclerosis has been demonstrated [10–16]. Bernard et al. demonstrated that circulating autoantibodies against BP180 represented the first intrinsic prognostic factor in BP [17]. It has been reported that coincidence of neurological diseases with BP significantly increases the risk of mortality in BP patients [18].

Studies have shown that patients who are affected by BP and neurological diseases have immunogenic BPAG1 in their skin and also in their brain and it has been suggested that alterations of the central nervous system in BP patients could expose the neural isoforms of BPAG1 or other BP antigens. Autoantibodies against these antigens in the brain can cross-react with antigens in the skin [10, 19] and this cross-reactivity between the epithelial and neuronal isoforms of BPAG1 can cause the neurological event [20]. However, this association has not been completely understood.

Some studies have suggested that neurological diseases can both be a predisposing factor and contribute to prognostic factors in BP [21, 22]. Therefore, the purpose of this study was to determine the prevalence of neurological diseases in BP patients in southern Iran and to compare it to a matched control group.

## 2. Materials and Methods

Medical histories of 87 biopsy-proven BP patients hospitalized in Faghihi Hospital, Shiraz, Iran, from March 2001 to March 2016 were reviewed. Age, sex, and history of neurological diseases including dementia, stroke, Parkinson's disease, epilepsy, and multiple sclerosis were recorded. Diagnosis of BP was made based on positive direct immunofluorescence (DIF) tests and H & E staining of skin biopsies.

The medical history and medications used by the patients were reviewed and the presence of any of the above-mentioned neurological diseases was recorded. In case of any ambiguity in the medical history, the patients were contacted and examined by a neurologist. The patients who had missing or incomplete records were excluded from the study.

The control group consisted of 184 individuals who were the visitors of the patients in the general surgery emergency ward of Faghihi Hospital between October 2015 and September 2016. Having BP or any other inflammatory skin diseases was the exclusion criterion for the control group. For each patient with BP, approximately two randomly chosen controls were matched according to age (±2 years) and gender. The control group was examined and if any sign or symptom of a neurological disease was observed, they were referred to a neurologist for better evaluation and diagnosis of the disease. The control group was suitable for this study because both the case and control groups had similar ethnicity.

All participants were required to fill out an informed consent form. This study was approved by the Ethics Committee of Shiraz University of Medical Sciences. Data were analyzed by SPSS statistical software version 19.0, using chi-square test and *t*-test. A *P* value ≤ 0.05 was considered as statistically significant.

## 3. Results

Eighty-seven patients who met the inclusion criteria were enrolled in the study. The mean age in the case group was 64.2 ± 18.1 years and in the control group it was 60.7 ± 21.7 years. The case group consisted of 41 male patients and 46 female patients, while there were 86 males and 98 females in the control group. No significant gender or age differences were found between the two groups (*P* values = 0.95, 0.19) (Table 1).

Out of the 87 patients with BP, 17 (19.5%) had at least one neurological disease. CVA was the most common neurological disease observed in 7 patients (8.0%) in the case group and 4 patients (2.1%) in the control group. The incidence of CVA was significantly different between BP patients and the control group (*P* = 0.02). Dementia was observed in 6 patients (16.8%) in the case group and 2 patients (1.1%) in the control group. The difference in the incidence of dementia was statistically significant between BP patients and the control group (*P* = 0.008). In this study, the incidences of Parkinson's disease (*P* = 0.830), epilepsy (*P* = 0.067), and multiple sclerosis (*P* = 0.326) between the two groups were not statistically significant (Table 2).

In the case group, 17 patients had at least one neurological disease and the mean age of these patients was 67.6 ± 19.6 years. The mean age in the case group without neurological disease was 63.3 ± 17.7 years. This was not statistically significant (*P* = 0.387).

In the control group, 13 patients had a neurological disease. The mean age in the subjects with neurological disease in the control group was 76.6 ± 9.9 years. The mean age in the control group subjects without any neurological disease was 59.5 ± 21.9 years. This difference was statistically significant (*P* = 0.006).

## 4. Discussion

Many studies have shown an association between BP and various neurological disorders, but there is a variation in the specific subset of these observed disorders. Many studies noted that amyotrophic lateral sclerosis is associated with BP [23–26]. Some studies have shown an association between multiple sclerosis and BP [27–30]. The French Study Group for Bullous Diseases demonstrated that there is an association between dementia, Parkinson's disease, and BP [31]. In a nationwide, registry-based study by Försti et al. between 1987 and 2013, 4524 patients with BP were compared to 36240 patients with BCC as their control group since BCC affects elderly people as does BP and is not an inflammatory disease, so it was regarded as a proper control group. They found that the most common neurological comorbidities in BP patients were cerebral infarction, dementia, and Alzheimer's disease [32].

In our study, patients with BP were significantly more likely to have CVA than the controls. This has been reported in previous studies [13, 15, 16, 19, 33, 34]. In some studies, dementia was the most prevalent neurological disorder in patients with BP [14, 35, 36], but the interesting point is that, in these studies, the mean age of BP patients was higher than the mean age in our case group. In the study that was done by Brick et al. in 2014, the mean age of the cases was 75.5 years and, in the study by Cordel et al., the mean age of the cases was 73.9 years. Kwan et al. conducted a retrospective case-control study on 43 patients with BP in 2015, and the mean age for the case group in that study was 79.4 years and dementia was the only neurological disease that had any statistically significant association.

Among our case group, 17 patients (19.5%) had at least one neurological disease (in some patients two or more neurological diseases existed together). Our study showed that the prevalence of neurological diseases in Iranian patients with BP was less than what has been reported in other countries, especially in European countries. This percentage was about 46% in a case-control study by Taghipour et al. in England [12], 36% in a study by Cordel et al. in France [14], and 55.8% in a study by Teixeira et al. in Portugal [15], but the subset of associated neurological diseases observed in our study were almost similar to those studies. Our findings were in accordance with the study of Daneshpazhooh et al. from Iran. The frequency of neurological diseases in their case group was 26.3%. They noted that stroke (17.5%) and dementia (5.6%) were significantly associated with BP [37].

## 5. Conclusion

The results of this study indicate an association between BP and neurological diseases. CVA was the most commonly observed neurological disorder in patients with BP in this study. The incidence of CVA and dementia in patients with BP compared to the control group was significantly higher.

## Figures and Tables

**Table 1:** Demographic characteristics of patients with BP and controls.

Variable	Cases	Controls	*P* value
Age, mean years	64.21 ± 18.1	60.74 ± 21.7	0.198
Sex			
Male	41 (47.1%)	86 (46.7%)	0.952
Female	46 (59.9%)	98 (53.3%)	

**Table 2:** Prevalence of neurological diseases in patients with BP and controls.

Neurologic disorder	Cases^a^ (*N* = 87)	Controls (*N* = 184)	*P* value
CVA	7 (8.04%)	4 (2.17%)	0.022
Dementia	6 (6.89%)	2 (1.08%)	0.008
Parkinson's disease	3 (3.44%)	4 (2.17%)	0.83
Epilepsy	4 (4.59%)	2 (1.08%)	0.067
Multiple sclerosis	1 (1.14%)	1 (0.54%)	0.326

BP: bullous pemphigoid; ^a^three patients with BP had more than 1 neurological disease.
